# Mesenchymal Stem/Stromal Cells May Decrease Success of Cancer Treatment by Inducing Resistance to Chemotherapy in Cancer Cells

**DOI:** 10.3390/cancers14153761

**Published:** 2022-08-02

**Authors:** Taja Železnik Ramuta, Mateja Erdani Kreft

**Affiliations:** Institute of Cell Biology, Faculty of Medicine, University of Ljubljana, 1000 Ljubljana, Slovenia; taja.zeleznik@mf.uni-lj.si

**Keywords:** mesenchymal stem/stromal cells, tumour microenvironment, tumour stroma, cancers, cancer therapy, drug resistance

## Abstract

**Simple Summary:**

Tumours consist of different cell types and an extracellular matrix, all of which together form a complex microenvironment. The tumour microenvironment plays a critical role in various aspects of tumour development and progression. Mesenchymal stem/stromal cells (MSCs) are multipotent stem cells that have a tri-lineage differentiation capacity and are one of the key stromal cells in the tumour microenvironment. Following the interaction with cancer cells, they are transformed from naïve MSCs to tumour-associated MSCs, which substantially affect tumour growth and progression as well as the development of chemoresistance in cancer cells. The aim of this review article is to provide an overview of studies that have investigated how MSCs affect the susceptibility of cancer cells to chemotherapeutics. Their results show that MSCs protect cancer cells from chemotherapeutics by influencing several signalling pathways. This knowledge is crucial for the development of new treatment approaches that will lead to improved treatment outcomes.

**Abstract:**

The tumour microenvironment, which is comprised of various cell types and the extracellular matrix, substantially impacts tumour initiation, progression, and metastasis. Mesenchymal stem/stromal cells (MSCs) are one of the key stromal cells in the tumour microenvironment, and their interaction with cancer cells results in the transformation of naïve MSCs to tumour-associated MSCs. The latter has an important impact on tumour growth and progression. Recently, it has been shown that they can also contribute to the development of chemoresistance in cancer cells. This review provides an overview of 42 studies published between 1 January 2001 and 1 January 2022 that examined the effect of MSCs on the susceptibility of cancer cells to chemotherapeutics. The studies showed that MSCs affect various signalling pathways in cancer cells, leading to protection against chemotherapy-induced damage. Promising results emerged from the use of inhibitors of various signalling pathways that are affected in cancer cells due to interactions with MSCs in the tumour microenvironment. These studies present a good starting point for the investigation of novel treatment approaches and demonstrate the importance of targeting the stroma in the tumour microenvironment to improve treatment outcomes.

## 1. Introduction

Mesenchymal stem/stromal cells (MSCs) are multipotent stem cells that have atri-lineage differentiation capacity, namely, into osteoblasts, chondrocytes, and adipocytes [1,2,3]. They express the surface markers CD73, CD105, and CD90 [1,4]. MSCs can be isolated from various sources, such as the bone marrow [5], adipose tissue [5], dental pulp [6], umbilical cord [7], placenta [8], endometrium [9], amniotic fluid [10], skeletal muscle tissue [11], liver tissue [5], and other tissues [1,2,12]. MSCs are capable of homing to various injury (inflammation) sites, where they support tissue repair, stem cell homeostasis, and immunomodulation [12,13,14]. As the tumour microenvironment is similar to the microenvironment of chronic wounds, it attracts MSCs, especially those derived from the bone marrow, adipose tissue, and umbilical cord [14]. The homing and migration of MSCs are mediated by tumour-cell-specific receptors, the extracellular matrix, and various inflammatory cytokines, chemokines, and growth factors (e.g., MCP-1, SDF-1, IL-8, TGF-β, and VEGF) [12,15,16,17]. Moreover, a pro-inflammatory toll-like receptor ligand LL-37 has been linked to tumour-homing, as various tumour types (e.g., ovarian, breast, and lung cancer) demonstrated the overexpression of LL-37, which acted as a proliferative signal and pro-angiogenic factor and promoted the migration of MSCs into the tumour microenvironment, while its inhibition resulted in reduced tumour growth [18].

Tumours are comprised of various cell types and extracellular matrix, which together form a complex microenvironment. It has been shown that the tumour stroma, which is comprised of MSCs, tumour vasculature, infiltrating inflammatory and immune cells, extracellular matrix, and fibroblasts, plays a crucial role in tumour initiation, progression, and metastasis [19,20]. Furthermore, MSCs are one of the crucial stromal cells in the tumour microenvironment, and their interactions with tumour cells and various other components of the tumour microenvironment lead to the transformation from naïve MSCs to tumour-associated MSCs [21,22]. The latter are implicated in tumour growth and progression as well as the development of chemoresistance in cancer cells [23,24,25]. Since the distinction between “naïve” and “tumour-associated” MSCs is not consistently listed in most studies, we will use the general term MSCs in this review unless otherwise stated. However, more attention needs to be paid to these specific stages of MSCs in the future.

In many patients, the initial response to anticancer agents is favourable and improves disease-free survival. However, many tumours eventually become resistant to therapy, leading to relapse and the recurrence of cancer. Hence, it is of the utmost importance to elucidate the mechanisms that contribute to chemoresistance, which will lead to the development of novel strategies to overcome this issue.

## 2. The Ambivalent Nature of MSCs in the Tumour Microenvironment

In recent years, numerous studies have investigated the properties of MSCs in the tumour microenvironment, which have been reported in detail elsewhere [21,23,25,26,27]. Hence, herein we will briefly describe the properties of MSCs and then focus on the effects of MSCs on chemotherapeutic agents in the tumour microenvironment.

MSCs present an integral part of the tumour microenvironment and largely [28] contribute to tumour progression and metastasis [29]. First, MSCs are recruited to the tumour microenvironment, where the direct interactions between the tumour cells, MSCs, and tumour-secreted factors lead to the development of a pro-tumourigenic subpopulation of MSCs, i.e., tumour-associated MSCs [21,30,31]. These contribute to tumour progression by (a) differentiating into other pro-tumourigenic cells in the tumour microenvironment (e.g., cancer-associated fibroblasts) [14,32,33], (b) suppressing the immune response, (c) promoting the epithelial-to-mesenchymal transition, (d) promoting angiogenesis, (e) enriching the cancer stem cell population [29,30,34,35,36], and (f) promoting tumour cell survival and metastasis [14,21,24,33,37].

When using naïve MSCs in in vitro and in vivo studies, researchers have shown that the cells possess tumour-promoting and tumour-suppressing effects. Several studies demonstrated the capability of MSCs to promote tumour survival and angiogenesis, enhance invasion and metastatic potential, and inhibit the apoptosis of cancer cells, thus supporting tumour growth [24,38,39,40,41,42]. MSCs have been shown to release factors such as VEGF, FGF-2, PDGF, HGF, BDNF, SDF-1α, IGF-1, IGF-2, TGF-β, TNFα IGFBP-2, LIF, M-CSF, MIP-2, IL-8, IL-6, and IFNγ that promote tumour survival, proliferation, migration, invasion [21,43,44,45,46,47], and angiogenesis [21,24,25,33,48,49,50,51,52]. Furthermore, it has been shown that cancer cells induce epigenomic mesenchymal-to-epithelial transition in MSCs, resulting in pro-tumorigenic functions of MSCs, which then serve as a metastatic driver/chaperone [28]. Interestingly, other cell types in the tumour microenvironment may also transform into MSC-like cells and drive tumour resistance to cytotoxic agents, which was demonstrated in the case of glioblastoma, as the Wnt-mediated endothelial cell transformation resulted in MSC-like cells [53]. 

In addition, MSCs promote the epithelial-to-mesenchymal transformation by secreting CCL5, which promotes the secretion of matrix metalloproteinases that loosen the extracellular matrix in the tumour microenvironment and consequently contribute to cancer cell motility [21,54]. MSCs also have immunosuppressive properties that facilitate cancer cells to escape immune surveillance. They secrete several mediators, e.g., TGFβ, TNFα, IFNγ, indoleamine 2,3-dioxygenase (IDO), IL-1β, IL-1α, IL-4, and IL-6, and interact with various immune cells, all leading to immunosuppression [25,55,56,57]. For example, MSCs promote CD8+ T-cell tumour immune exclusion and decrease the response to anti-PD-L1 immune checkpoint inhibitor due to the secretion of the chemokines CCL2, CX3CL1, and TGFβ1 [58].

On the other hand, in some cases, MSCs are also capable of inhibiting tumour growth, which has been shown, for example, in the in vitro and in vivo models of breast cancer, Kaposi’s sarcoma, liver cancer, and melanoma [1,59,60,61,62]. The MSCs suppress tumour growth by inhibiting the signalling pathways associated with proliferation (e.g., STAT3, AKT, PI3K, and Wnt signalling pathways), cell cycle progression [13,52,60,63,64,65,66], the inhibition of apoptosis, and the suppression of angiogenesis [13,25,41,52,59,60,61,62,63,67,68,69,70,71,72]. The anticancer properties of MSCs have been reported in more detail elsewhere [19,73,74].

Another important point to consider is the variation in the structure of the tumour microenvironment among different cancers, as it has been shown that tumour microenvironments differ in terms of cell types and the composition of the extracellular matrix [75,76,77]. Moreover, crosstalk between MSCs and other cell types in the tumour microenvironment and certain properties of the microenvironment (e.g., pH, O_2_ concentration, and ion gradients) importantly affect MSC development and characteristics and the heterogeneity of the population [73,78]. To conclude, the ambivalent nature of MSCs was well-demonstrated by Ramasamy et al. (2007). They showed that naïve MSCs inhibit the proliferation of cancer cells in vitro, but the injection of MSCs into nude mice promotes tumour growth [38]. While the discrepancies in the effect of MSCs are partly due to the heterogeneity of the MSC populations and the different experimental conditions [13,19], these findings also underline the importance of considering the contribution of non-cancer cells in the tumour microenvironment to disease development and the response to treatment.

## 3. MSCs Promote Resistance of Cancer Cells to Chemotherapeutic Agents

**Figure 1:**
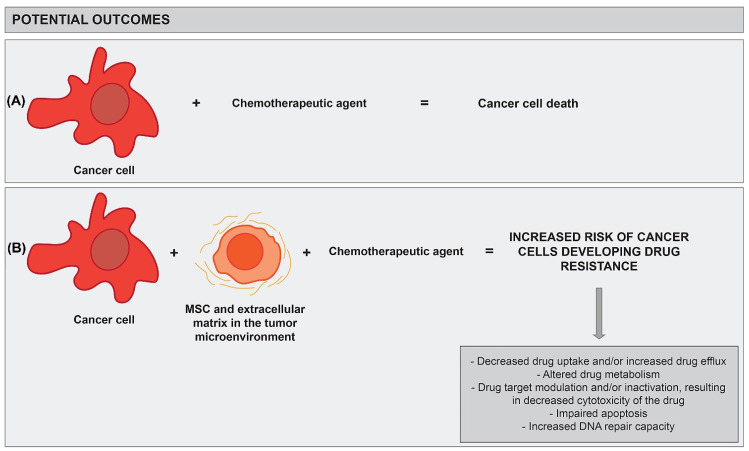
MSCs in the tumour microenvironment affect the success of anticancer therapy. There are two potential outcomes: (**A**) chemotherapeutic agents induce cell death in cancer cells or (**B**) the interaction of cancer cells, treated with a chemotherapeutic agent, with MSCs and the extracellular matrix in the tumour microenvironment may result in an increased risk of cancer cells developing drug resistance.

Drug resistance may result from genetic changes in cancer cells (intrinsic resistance) or from the tumour microenvironment (extrinsic resistance) [29,79]. The latter promotes drug resistance by (1) preventing drug penetration into the tumour due to changes in the composition of the extracellular matrix, (2) by secreting protective cytokines, or (3) by changing gene expression in tumour cells, which leads to decreased cytotoxicity of chemotherapeutics [79,80,81,82,83] (Figure 1). As chemotherapeutics induce damage in the tumour microenvironment, this results in a stress response in MSCs and cancer-associated fibroblasts, which secrete various growth and anti-apoptotic factors (e.g., IL-6, IL-7, IL-8, CXCL12, and VEGF) that promote cell survival, proliferation, invasion, and the metastasis of cancer cells [84,85,86,87,88,89,90,91]. Furthermore, the latest studies show that MSCs also play an important role in the development of drug resistance, which is the main focus of this review article. 

To investigate this topic in detail, we performed a comprehensive literature search using PubMed to identify articles investigating the effects of MSCs on chemotherapy resistance. We used the following search terms: ((“mesenchymal stem”) OR (“mesenchymal stromal”) OR (“MSCs”) AND (“chemotherapy”) AND (“resistance”)) and included only articles in which these terms appeared in the title and/or the abstract. Only full-text articles that were published in the English language between 1 January 2001 and 1 January 2022 were included. We identified 224 articles through database research, and after the removal of duplicates and review articles, editorials, and conference proceedings, there were 155 articles that were assessed in detail. Next, we excluded the articles that were irrelevant for the topic of this review article. The remaining 42 articles were included in the analysis, and their conclusions are presented in the following chapters and Table 1.

### 3.1. Blood Cancers

#### 3.1.1. Acute Lymphoblastic Leukaemia

To investigate the mechanism of resistance to chemotherapy-induced apoptosis in B-cell acute lymphoblastic leukaemia (B-ALL), Yu et al. (2020) focused on the protein tyrosine phosphatase Shp2, which is highly expressed in bone marrow MSCs of patients. The plasmid-induced Shp2 activation in MSCs led to an increase in chemotherapy-resistant cancer cells in the in vitro and in vivo settings. Moreover, they demonstrated that Shp2 activation in MSCs upregulates vascular cell adhesion molecule 1 (VCAM-1) expression by increasing the PI3K/AKT phosphorylation level. Moreover, the inhibition of very late antigen-4 (VLA-4) reversed the chemotherapy resistance, which indicates that targeting VCAM-1/VLA-4 signalling may have clinically relevant value [92]. Its importance was also demonstrated by Jacamo et al. (2014), who showed that VCAM-1/VLA-4 signalling plays an integral role in the activation of NF-κB in leukaemia and stromal cells, which is also crucial for promoting the chemoresistance of cancer cells [93].

The CXCR/CXCL12 axis plays a crucial role in the homing of hematopoietic stem cells and ALL cells to the bone marrow microenvironment, and the overexpression of CXCR4 in ALL patients is associated with disease progression and poor prognosis [94,95]. Wang et al. (2020) cultured cells from patients with relapsed/refractory ALL with MSCs, which resulted in a higher expression of CXCR4 and CXCL12 in the presence of the chemotherapeutic agent vincristine. However, when the CXCR4 antagonist AMD3100 was added, the expression of CXCR4 and CXCL12 was reduced, which showed that blocking the CXCR4/CXCL12 axis results in sensitizing the relapsed/refractory ALL cells to chemotherapeutic agents [96]. Moreover, Pillozzi et al. (2019) identified the novel peptide 4-1-17 and the small molecule 8673 for targeting the CXCR4/CXCL12 axis, the use of which, in an in vitro setting, induced a pro-apoptotic effect that was not reduced by the presence of MSCs. The treatment using both novel tools was capable of overcoming the MSC-induced resistance to the chemotherapeutic agent cytarabine in ALL-derived cell lines [97]. 

The Wnt signalling pathway regulates cell proliferation, survival, differentiation, and migration and is also involved in MSC-mediated drug resistance [98]. Yang et al. (2013) grew co-cultures of ALL cells and MSCs and found that MSCs protect cancer cells from spontaneous cytarabine-induced apoptosis, which results from the decreased cleavage of apoptotic proteins and the modified expression of various cell-cycle-related proteins. Moreover, they also demonstrated that MSCs activate the Wnt signalling pathway in ALL cells. Importantly, by using a Wnt signalling inhibitor, the susceptibility of ALL cells to chemotherapy was improved [99].

In the case of T-cell ALL, MSCs contribute to the development of chemoresistance by accepting mitochondria from cancer cells that underwent chemotherapy-induced intracellular oxidative stress. The transfer is mediated by tunnelling nanotubes, and by inhibiting the mitochondria transfer using cytochalasin D, the capacity of MSCs to protect cancer cells from a chemotherapeutic agent decreased. Interestingly, in the co-culture setting, most of the cancer cells adhered to MSCs, and researchers showed that the adhesion is ICAM-1-mediated and contributes to MSC-induced chemoresistance [100].

#### 3.1.2. Acute Myeloid Leukaemia 

Various studies have demonstrated that MSCs protect cancer cells from chemotherapy-induced genotoxicity. For example, Gynn et al. (2021) showed that primary MSCs in co-culture with leukaemic HL-60 cells are sensitized to cytarabine-induced genotoxicity, while leukaemic cells remain protected from the agent. Moreover, primary MSCs from patients who had undergone chemotherapy show reduced survival and increased genotoxicity in comparison to patients’ MSCs at diagnosis, and MSCs also retain the long-term genotoxic and functional damage following exposure to cytarabine [101]. Moreover, patient-derived data show that the poor therapy response in patients with AML correlates with increased quiescence. The phenomenon was also confirmed in an in vitro study in which co-cultures of MSCs and AML cells (in direct contact and in the transwell setting) showed that MSCs protect cancer cells from the apoptosis induced by the chemotherapeutic agent cytarabine by enriching the population of quiescent cells [102].

MSCs secrete TGF-β1 and CXCL12, which are the key factors in the bone marrow niche that govern hematopoietic cell proliferation and survival; TGF-β1 maintains quiescent hematopoietic stem cells, while CXCL12 promotes their homing and anchoring in the niche [103,104]. The co-culture of bone marrow MSCs and AML cells led to a higher proliferation capacity of AML cells in comparison to a monoculture. Moreover, the blockade of TGF-β1 increased the proliferation and chemosensitivity of AML cells, while the addition of a CXCR4 antagonist resulted in anti-proliferative effects, but it did not affect the susceptibility of AML cells to the chemotherapeutic cytarabine [105]. 

Increased aldehyde dehydrogenase (ALDH) activity in AML cells is associated with greater tumourigenicity and chemotherapy resistance. Yuan et al. (2020) demonstrated that the ratio of ALDH-positive cells increased when cancer cells were incubated with MSCs, and further analysis revealed that the TGF-β1-regulated gene signature was activated in cancer cells when they were in co-culture with MSCs. Further data demonstrated that the knockdown of TGF-β1 in MSCs inhibited stroma-induced ALDH activity in cancer cells, while on the other hand, the treatment with recombinant TGF-β1 induced ALDH activity in cancer cells. Moreover, the TGF-β1-induced ALDH2 expression in AML cells was regulated by the non-canonical pathway through the activation of p38. Importantly, the use of two inhibitors of ALDH2 led to the sensitization of AML cells to chemotherapy, even in the presence of MSCs [106]. 

Dong-Feng et al. (2014) investigated the role of the connection between the leukaemic stem cells and the hematopoietic microenvironment, mediated by the thrombopoietin/c-MPL pathway, in developing chemoresistance. They showed that when in co-culture with osteoblasts differentiated from bone-marrow-derived MSCs from acute myeloid leukaemia (AML) patients, cancer cells secreted more thrombopoietin and were more resistant to the chemotherapeutic daunorubicin than when co-cultured with osteoblasts derived from normal control MSCs. Hence, their results indicate that a high level of thrombopoietin/c-MPL signalling may contribute to increased chemoresistance in AML [107].

In 2014, Xia et al. demonstrated that bone-marrow-derived MSCs protect AML cells from chemotherapy-induced apoptosis via the c-Myc-dependent pathway [108]. Next, in 2017, the same research group showed that in the co-culture with MSCs, AML cells decreased the expression of miRNA-494, which is one of the c-Myc regulators, and its downregulation is associated with poor prognosis in AML patients. Moreover, the activation of miR-494 in AML cells in combination with mitoxantrone in vitro led to suppressed proliferation and induced apoptosis and in vivo resulted in suppressed tumour growth and prolonged survival in mice. Therefore, their results indicated that miR-494 suppresses drug resistance in AML cells by downregulating c-Myc [109]. 

O’Reilly et al. (2018) showed that bone-marrow-derived MSCs promote the resistance of AML cells to the chemotherapeutic agents cytarabine and daunorubicin and to BH_3_ mimetics. One of the most studied mechanisms of chemoresistance is the activation of pro-survival signal transduction and the induction of anti-apoptotic Bcl-2 proteins [110,111]. Moreover, another anti-apoptotic protein, myeloid cell leukemia-1 (Mcl-1) protein, acts as a master regulator of apoptosis [112]. The results of O’Reilly et al. show that chemotherapy resistance is mediated by MSC-induced Mcl-1 expression over Bcl-2 and/or Bcl-X_L_ in AML cells, and the inhibition of Mcl-1 restores sensitivity to BH_3_ mimetics, while the combined inhibition of Bcl-2/Bcl-X_L_ and Mcl-1 restores susceptibility to cytarabine and daunorubicin [110].

Prostaglandin E2 (PGE2) suppresses apoptosis in multiple cell types, and it has been shown that malignant cells release IL1β, which induces PGE2 synthesis in MSCs, which is followed by the activation of β-catenin signalling and the induction of the cancer stem cell phenotype. PGE2 is an enzymatic product of Cox-2, and both proteins are involved in inflammation and cancer, although the role and regulation of PGE2 in the case of AML are not well-documented [113,114]. Carter et al. (2016) showed that apoptosis repressor with caspase recruitment domain (ARC) protein induces the expression of IL-1β in cancer cells when they are in co-culture with MSCs and increases the expression of CCL2, CCL4, and CXCL12 in MSCs. Hence, ARC confers chemoresistance by controlling the interactions between cancer cells and their microenvironment through an NFκB/IL1β signalling network. Importantly, the inhibition of IL1β suppressed cancer cell migration and increased their susceptibility to chemotherapeutics [115]. Next, the same research group showed that co-cultures of AML cells and MSCs promoted COX-2 expression in MSCs and PGE-2 production. The latter induces the expression of β-catenin, which regulates the protein ARC and promotes chemoresistance in AML cells. Furthermore, the study showed that ARC is an integral component of the IL1β/COX-2/PGE2/β-catenin circuit and that ARC, which is regulated by β-catenin, mediates AML-stromal interactions. Importantly, the inhibition of β-catenin led to a decrease in ARC and sensitized AML cells to chemotherapy [114].

#### 3.1.3. Chronic Lymphocytic Leukaemia 

In 2012, Nwabo Kamdje et al. investigated the role of stromal-cell-mediated signalling in chronic lymphocytic leukaemia resistance to chemotherapy. They showed that when cancer cells were in co-culture with autologous or allogeneic bone-marrow-derived MSCs, the latter protected cancer cells from spontaneous or chemotherapy-induced apoptosis. Moreover, following treatment with a combination of anti-Notch-1, anti-Notch-2, and anti-Notch-4 antibodies or γ-secretase inhibitor XII, cancer cells had increased susceptibility to chemotherapeutics [116]. Similarly, Kurtova et al. (2009) and Severin et al. (2019) demonstrated that human and murine MSCs are effective in protecting chronic lymphocytic leukaemia cells from fludarabine-, dexamethasone-, cyclophosphamide-, and ibrutinib-induced apoptosis. Interestingly, Kurtova et al. showed that the MSCs triggered this effect only when in cell–cell contact with cancer cells but not when cultivated in a transwell setting [117,118]. 

#### 3.1.4. Multiple Myeloma

Yang et al. (2017) investigated the effect of bone-marrow-derived MSCs from patients with multiple myeloma on cell lines of multiple myeloma. They showed that MSCs decreased melphalan- or doxorubicin-induced cell cycle arrest and apoptosis. Moreover, MSCs promoted the expression of autophagy-related genes in cancer cells, leading to the activation of autophagy and subsequently NF-κB signalling [119], which promotes the proliferation, survival, and drug resistance of cancer cells [120]. When NF-κB signalling was inhibited, MSCs no longer had a protective effect on cancer cells. Using autophagy inhibitors (chloroquine and 3-methyladenine), it was shown that treatment suppressed the phosphorylation and consequent degradation of NF-κB inhibitor (I-κBα), which prevented the resistance of cancer cells to chemotherapeutic agents [119]. Furthermore, it has been shown that bone-marrow-derived MSCs, together with the non-cellular components, promote protective endoplasmic reticulum stress in multiple myeloma cells, thus bolstering their resistance to melphalan and bortezomib [121]. 

### 3.2. Glioma

The conditioned medium of glioma-associated MSCs has been shown to induce the overexpression of FOXS1 in glioma cells, which leads to the resistance of cancer cells to the chemotherapeutic agent temozolomide. Interestingly, the researchers identified two subpopulations of glioma-derived MSCs that play distinct roles in tumour progression. The CD90^high^ population of MSCs increases glioma cell proliferation, migration, and adhesion, while the CD90^low^ population of MSCs is involved in the development of glioma cell resistance to chemotherapy [122,123]. 

On the other hand, de Castro et al. (2017) showed that a conditioned medium from Wharton’s jelly-derived MSCs promoted the viability, proliferation, and migration of glioblastoma cell lines, but it did not significantly affect the susceptibility of cancer cells to temozolomide [124]. 

### 3.3. Neuroblastoma

Lifshitz et al. (2017) obtained the conditioned medium from bone-marrow-derived MSCs from patients with neuroblastoma and applied it to the neuroblastoma cell line CHLA-255. They showed that MSCs promote the expression of sphingosine-1-phosphate receptor 1 (S1PR1) expression in cancer cells as well as the phosphorylation of its downstream molecules JAK2 and STAT3 [125]. This leads to increased cell proliferation, the suppression of apoptosis, improved cell survival, and chemoresistance [126,127]. Next, they prepared an S1PR1 knockdown in neuroblastoma cells by shRNA, which enhanced chemotherapeutic agent etoposide-induced apoptosis in neuroblastoma cell lines. Similarly, the treatment of neuroblastoma cell lines with an antagonist of S1PR1 significantly increased the susceptibility of cancer cells to etoposide. On the other hand, the overexpression of S1PR1 protected the cancer cells from chemotherapy-induced apoptosis by activating JAK-STAT3 signalling, supporting the claim that S1PR1 plays an important role in chemoresistance [125]. 

### 3.4. Oral Squamous Cell Carcinoma

Receptor tyrosine kinases, such as the platelet-derived growth factor receptor (PDGFR), play a crucial role in promoting cell growth and proliferation [128]. In the case of oral squamous cell carcinoma (OSCC), it was shown that in the co-culture of OSCC cells and MSCs the latter activated a PDGF-AA/PDGFR-α autocrine loop, which led to reduced cancer cell sensitivity to cisplatin due to alterations in apoptosis. The authors concluded that the crosstalk between cancer cells and MSCs is at least in part mediated by the activation of PDGFR-α/AKT signalling pathways [129].

### 3.5. Head and Neck Cancers

The resistance of cancer cells to chemotherapeutics as a result of an interaction with MSCs is also induced in an epigenetic manner. This was demonstrated by Liu et al. (2021), who showed that the presence of MSCs supported the resistance of head and neck cancer lines (SCC-25, HSC-2) to paclitaxel by affecting the expression of genes that have been implicated in drug resistance (e.g., GDF15, CXCL11, TFP1-2, POSTN, IGFBP5, and DAPK1). The effect of MSCs on the drug resistance of cancer cells lasted as long as 30 days after the exposure of cancer cells to MSCs, while the largest effect was recorded at 3 days after exposure to MSCs [130]. 

Moreover, MSCs had a protective effect on head and neck cancer cell lines in the presence of paclitaxel, as the co-culture led to the decreased expression of cleaved-caspase 3 and cleaved-PARP-1 in cancer cells, which is associated a with lower level of drug-induced apoptosis [130]. 

### 3.6. Breast Cancer

Ullah et al. (2019) injected bone-marrow-derived MSCs and breast cancer cells into NOD/SCID mice and observed that xenograft tumours grew smaller in comparison to tumours induced by breast cancer cells alone but exhibited resistance to chemotherapeutic agents. Moreover, they showed that the chemoresistance was associated with an increased expression of tetraspanins (CD9 and CD81) and the drug resistance proteins BCRP and MDR1. Their findings showed that crosstalk between MSCs and cancer cells can be attributed to CD9, the overexpression of which is associated with a higher risk of invasion and metastasis as well as the promotion of the expression of BCRP and MDR1, which is mediated via CCL5, CCR5 and CXCR12 [131]. Next, Yeh et al. (2017) treated the MDA-MB-231 breast cancer cell line with the conditioned medium from adipose-derived MSCs isolated from breast cancer patients. They demonstrated that MSCs secreted CXCL1, which promoted doxorubicin resistance. MSCs downregulated miR-106a expression, which enhanced the expression of ATP-binding cassette transporters (ABCG2) through which doxorubicin is removed from the cell [132]. 

Luo et al. (2020) demonstrated that exosomes secreted by MSCs promote chemoresistance in breast cancer by enhancing the miR-21-5p-mediated expression of S100A6, a chemoresistance gene. Moreover, doxorubicin induced the expression of miR-21-5p, which was necessary for the expression of the S100A6 gene in cancer cells. However, silencing miR-21-5p expression in cancer cells resulted in cancer cells being susceptible to doxorubicin [133].

Lu et al. (2017) co-cultured breast cancer cells (MCF-7/ADR cell line) with adipose-derived MSCs and investigated the effect of MSCs on the expression of C-terminal Src kinase-binding protein (Cbp), which is a transmembrane adaptor protein that regulates Src family kinase activities. The presence of MSCs induced the increased expression of Cbp and increased cell proliferation and chemoresistance. Using RNA interference, the presence of Cbp in breast cancer cells was depleted, which led to the inhibition of cell proliferation and invasion. Moreover, in these conditions, the use of doxorubicin induced the suppression of tumour growth. The silencing of Cbp was followed by the inhibition of the expression of phosphoryl Src, AKT, and mTOR signals. Therefore, these results indicate that the PAG1/Cbp pathway is involved in breast cancer tumour progression and acquired chemoresistance and that the PAG1/Cbp pathway modulates MSC-promoted tumourigenesis via the Src and AKT/mTOR pathways [134].

Barneh et al. (2018) demonstrated that valproic acid, which is already being used in the clinic for the treatment of seizures and bipolar disorder [135], inhibits the protective effects of MSCs against chemotherapy in breast cancer. Using the proteomic dataset, the research group showed that secreted cytokines from MSCs activated various signalling pathways in cancer cells, including the JAK-STAT, PI3K, MAPK, and TGFβ signalling pathways, which lead to the activation of NF-kB transcription factor. Next, they showed that valproic acid affects the expression of 34 genes within the identified pathways, and in vitro experiments confirmed that valproic acid inhibits NF-kB activation in cancer cells. Moreover, the patients taking oral valproic acid demonstrated decreased expression of antioxidant enzymes, which led to increased oxidative stress in cancer cells. Hence, valproic acid hinders the protective role of MSCs by inhibiting the mechanisms allowing the adaptation of cancer cells to oxidative stress [136]. 

### 3.7. Lung Adenocarcinoma 

Wang et al. (2020) showed that, in the tumour microenvironment of lung adenocarcinoma, bone-marrow-derived MSCs produced molecules associated with adipocytes, which promoted resistance to the chemotherapeutic agent erlotinib in cancer cells by activating the insulin-like growth factor 1 receptor (IGF-1R). The MSCs in the hypoxic tumour microenvironment produced leptin, which is capable of inducing erlotinib resistance in lung adenocarcinoma cells through the activation of IGF-R1 signalling in the absence of IGF-1 in hypoxia. On the other hand, IGF binding protein 2 (IGFBP2) counteracted the activation of IGF-1 and induced erlotinib resistance by activating IGF-1R signalling in an IGF-independent manner. Moreover, the inhibition of IGF-1R with the inhibitor BMS754807 reversed the erlotinib resistance [137].

### 3.8. Gastric Cancer

Gastric-cancer-associated MSCs promote the resistance of gastric cancer cells to chemotherapy through the secretion of IL-8 and the consequent induction of PD-L1 [138]. The overexpression of PD-L1 affects the therapeutic efficacy of chemotherapy and is associated with a shorter survival period for patients [139]. Importantly, the sensitivity of gastric cancer cells to chemotherapy was enhanced when PD-L1 was blocked [138]. Another study showed that the co-culture of gastric cancer cells with MSCs induced the long non-coding RNA (lncRNA) HCP5 (histocompatibility leukocyte antigen complex P5), which drove fatty acid oxidation through the miR-3619-5p-AMPK-PGC1α/CEBPB axis and resulted in the promotion of stemness and the induction of chemoresistance [140]. Moreover, He et al. (2019) demonstrated that MSCs promote the stemness and chemoresistance of gastric cancer cells through fatty acid oxidation in vitro and in vivo. The MSCs secrete TGF-β1, which activates SMAD2/3 and induces long non-coding RNA (lncRNA) MACC1-AS1 expression in cancer cells. MACC-AS1 promotes chemoresistance by antagonizing miR-145-5p. Importantly, the inhibition of fatty acid oxidation by the inhibitor etomoxir attenuated the MSC-induced chemoresistance in vivo and therefore underlined the importance of targeting fatty acid oxidation [141]. 

In 2015, Ji et al. showed that MSC-derived exosomes significantly increased the chemoresistance of gastric cancer cells to 5-fluorouracil, resulting in decreased apoptosis and the increased expression of MDR, MRP, and LRP proteins, which are associated with multidrug resistance, leading to an enhanced capability of cancer cells to expel chemotherapeutics. In cancer cells, the exosomes of MSCs triggered the activation of calcium/calmodulin-dependent protein kinases (CaM-Ks) and the Raf/MEK/ERK cascade. Importantly, the tumour-promoting role of MSC-derived exosomes in chemoresistance was inhibited by blocking the CaM-KS/Raf/MEK/ERK pathway [142].

In 2016, Ji et al. showed that bone-marrow-derived MSCs increase drug resistance in CD133-expressing gastric cancer cells, which have been shown to possess cancer stem cell properties. Moreover, MSCs triggered the activation of the PI3K/AKT pathway in CD133-positive cells, resulting in decreased apoptosis and increased chemoresistance of CD133-positive cells due to the upregulation of Bcl-2 and the downregulation of BAX. Importantly, blocking the PI3K/AKT pathway prevented the promotion of chemoresistance, and they also showed that MSCs had no effect on drug resistance in CD133-knockdown cells, highlighting the involvement of CD133 in this process [143]. 

### 3.9. Pancreatic Adenocarcinoma 

Timaner et al. (2018) showed that MSCs, which homed to pancreatic adenocarcinoma and were located in close proximity to tumour-initiating cells, promoted the enrichment of tumour-initiating cells in vitro and enhanced tumour growth in vivo. These effects were due to the secretion of CXCL10 by gemcitabine-treated MSCs, which led to activation of the CXCL10-CXCR3 axis and subsequently to the activation of the STAT3 signalling pathway in tumour-initiating cells. Importantly, when the tumour-initiating cells were treated with gemcitabine and, at the same time, the CXCL10-CXCR3 axis was disrupted by the treatment with nanovesicles that contained CXCR3 antagonist, the treatment delayed tumour regrowth. Interestingly, they also showed that the MSC-dependent accumulation of tumour-initiating cells depends on the cell type, as this effect occurred in pancreatic adenocarcinomas but not in lung cancer, although both tumours express CXCR3 [144]. 

### 3.10. Genitourinary Cancers

Yu et al. (2021) showed that MSCs reduce the sensitivity of castration-resistant prostate cancer to docetaxel. Interestingly, the co-culture of cancer cells with MSCs in the presence of docetaxel led to an increase in autophagy activation, but the application of the autophagy inhibitor effectively reversed the resistance to docetaxel. Moreover, the increase in autophagy activation in the presence of docetaxel depended on TGF-β1 secretion, and the inhibition of TGF-β1 secretion increased the sensitivity of cancer cells to docetaxel [145].

Maj et al. (2016) investigated the effect of amniotic-fluid-derived MSCs and adipose-derived MSCs on the susceptibility of the 786-O human renal carcinoma and T24 bladder carcinoma cell lines to ciprofloxacin. They demonstrated that the MSC-derived conditioned medium reduced the sensitivity of cancer cells to ciprofloxacin and that the viability of cancer cells was 2.4–2.6-times higher when cells were incubated with ciprofloxacin in the presence of MSC-derived conditioned medium than in the presence of standard culture medium [146]. Similarly, conditioned medium from adipose MSCs reduced cisplatin-induced apoptosis in epithelial ovarian cancer cells, which resulted in cisplatin resistance and the increased viability of cancer cells [147]. 

Furthermore, Coffman et al. (2016) isolated cancer-associated MSCs from patient-derived tissue from patients with ovarian cancer. They demonstrated that Hedgehog secreted by ovarian cancer cells induces BMP4 expression in MSCs, which leads to the increased expression of Hedgehog in ovarian cancer cells, indicating a positive feedback loop [148]. Hedgehog signalling is crucial in ovarian cancer, as it is associated with increased chemoresistance and decreased survival [149,150]. Next, using a Hedgehog inhibitor or a BMP4-blocking antibody resulted in a decreased level of BMP4 derived from MSCs, which reversed resistance to cisplatin [148]. 

### 3.11. Liver Cancer

Han et al. (2014) treated hepatocellular carcinoma cells (SMMC-7721, Hep-G2 cell lines) with the conditioned medium derived from MSCs stimulated with IFN-γ and/or TNF-α. They demonstrated that in the presence of the MSC-derived conditioned medium the cancer cells underwent autophagy, which protected them against the cell toxicity of chemotherapeutics. However, when treated with an autophagy inhibitor, the cancer cells became susceptible to the chemotherapeutic agent 3-methyladenine. Additionally, they also showed that the stimulation of MSCs with IFN-γ and TNF-α promoted the expression of TGF-β in MSCs and that the upregulation of TGF-β correlates with chemoresistance in cancer cells. Moreover, the inhibition of TGF-β expression in MSCs diminished the capability of MSCs to induce autophagy, which resulted in diminished chemoresistance in cancer cells [151]. 

### 3.12. Bone Cancers

Tu et al. (2016) obtained the conditioned medium from bone-marrow-derived MSCs and used it for the treatment of the human osteosarcoma cell lines Saos-2 and U2-OS. They showed that MSCs had a protective role, as the presence of MSC-derived conditioned medium protected cancer cells from apoptosis when cultured with the chemotherapeutic agents doxorubicin or cisplatin. STAT3 activation by IL-6 has been shown to regulate MSC-induced chemoresistance, and the blockage of STAT3 activation (with AG490, a JAK2 inhibitor) improved the susceptibility of cancer cells to chemotherapeutic agents. Furthermore, when osteosarcoma mice were injected with MSCs and Saos-2 cells, the inhibition of STAT3 improved survival by suppressing tumour growth and increasing their susceptibility to doxorubicin. The role of STAT3 in chemoresistance was further supported by data obtained from osteosarcoma samples from patients, namely, increased expression of p-STAT3, MRP (multidrug resistance protein), and MDR-1 (P-glycoprotein) correlated with chemoresistance [152].

**Table 1:** Overview of studies investigating the effects of MSCs on chemotherapeutic-agent-treated cancer cells. Bold text indicates the crucial effects of MSCs on chemotherapeutic-agent-treated cancer cells.

Type of Cancer	Source of MSCs	In Vitro Model	In Vivo Model	Chemotherapeutic Agent	Effects of MSCs on Chemotherapeutic-Agent-Treated Cancer Cells	References
**ALL**	Human umbilical-cord-derived MSCs	Human patient-derived ALL cells	N/A	Vincristine	MSCs mediated vincristine resistance by **blocking the CXCR4/CXCL12 axis**.	[96]
	Human bone-marrow-derived MSCs from ALL patients and healthy volunteers	Human CCRF-SB B lymphoma cell line	NOD/SCID mice	Vincristine	Protein tyrosine phosphatase 2 (Shp2) activation in the BMSC **upregulates VCAM-1 expression** through activating the PI3K/AKT signalling pathway, which thereby interacts with VLA-4 in B-ALL cells to resist vincristine.	[92]
	Human bone-marrow-derived MSCs	Human B-cell precursor-ALL cells and AML cells; human normal Epstein–Barr-virus-infected B lymphocytes	N/A	Peptide 4-1-17, small molecule 8673, cytarabine, doxorubicin	**Peptide 4-1-17 and small molecule 8673 inhibit leukaemia cell proliferation and induce a pro-apoptotic effect that is not reduced by the presence of MSCs**. The combined treatment with 4-1-17 and 8673 has a stronger pro-apoptotic effect, particularly on cells cultured on MSCs in normoxic and hypoxic conditions, and can overcome MSC-induced resistance to cytarabine.	[97]
	Human bone-marrow-derived MSCs	Human T-cell ALL cell line Jurkat; human primary T-cell ALL cells	N/A	Cytarabine	The chemotherapeutic drug causes intracellular oxidative stress in Jurkat cells. Jurkat cells transfer mitochondria to MSCs via tunnelling membrane nanotubes but **receive few mitochondria from MSCs, resulting in chemoresistance**.	[100]
	Human bone-marrow-derived MSCs from healthy volunteers	Human leukaemia pre-B ALL cells; human primary ALL mononuclear cells	N/A	Everolimus	**BM stromal cells enhance HIF-1α expression under hypoxia, leading to an HIF-1α-dependent upregulation of glucose transport** and a switch to glycolytic metabolism in leukaemic cells and primary ALL blasts. Downregulation of HIF-1α expression or blockade of mTOR signalling with everolimus promoted chemosensitivity.	[153]
	Human bone-marrow-derived MSCs, human MSC line HS-5, murine MSC line M2-10B4	The human leukaemia cell lines Reh, RS4;11, and SEMK2	NOD/SCID mice	Cytarabine	**MSCs induce activation of the Wnt pathway** in ALL cells, and this activation contributes to the survival of ALL cells. Blocking the Wnt pathway with the β-catenin inhibitor XAV939 partially overcame the MSC-mediated cytarabine resistance of ALL cells both in vitro and in vivo.	[99]
	Human bone-marrow-derived MSCs from patients with ALL or AML	The human leukaemia cell lines OCI-AML3, Reh, NALM6-luciferaseCopGFP, and RS4;11	N/A	Vincristine, doxorubicine, cytarabidine	**The blockade of NF-κB activation via chemical agents or the overexpression of the mutant form of the inhibitor κB-α (IκBα) in BM-MSCs markedly reduced the stromal-mediated chemotherapeutic drug resistance in leukaemia cells** in vitro and in vivo. An in vivo model of the human leukaemia BM microenvironment also illustrated a direct link between NF-κB activation and stromal-associated chemoprotection.	[93]
**AML**	Human bone-marrow-derived MSCs from patients	Leukaemic cell line HL-60	N/A	Cytarabine	Primary mesenchymal stromal cells in co-culture with leukaemic HL-60 cells are sensitized to cytarabine-induced genotoxicity, while leukaemic cells are protected. **Malignant HSCs and MSCs bidirectionally modulate genotoxicity**.	[101]
	Human bone-marrow-derived MSCs from healthy volunteers	Leukaemic cell line HL-60	NOD/SCID mice	Cytarabine	**BM stromal cells induce ALDH activity in AML cells** through increased expression of the ALDH2 isoform. **BM-MSCs secrete TGF-β1, which exerts its effect through a noncanonical/p38-dependent signalling mechanism, leading to a stem-like phenotype in AML cells**. Inhibition of downstream targets of this pathway, such as p38 MAPK, inhibits ALDH activity in AML cells. ALDH2 inhibition sensitizes AML cells to standard cytarabine chemotherapy in vitro. However, these findings were not validated in vivo.	[106]
	Human bone-marrow-derived MSCs from healthy volunteers	Human leukaemia cell line OCI-AML3; human primary cells from AML patients or healthy volunteers	NSG mice	Cytarabine	Cox-2 expression and PGE2 generation are ARC/IL1β-dependent. The apoptosis repressor with caspase recruitment domain (ARC) protein, regulated by β-catenin, is an integral component of an IL1β/PGE2/β-catenin circuit. **Cox-2/PGE2, regulated by ARC and induced by AML-MSC co-culture contributes to MSC-mediated chemoprotection in AML**.	[114]
	Human bone-marrow-derived MSCs	Human leukaemia cell lines OCI-AML2, OCI-AML3, HL-60, ML-1, and Molm-13	N/A	BH3 mimetics, cytarabine, and daunorubicin	**Bcl-2/Bcl-XL and Mcl-1 act redundantly as effectors of BMM-mediated AML drug resistance**, highlighting the potential of Mcl-1 suppression to reverse BMM-mediated drug resistance in the leukaemic stem cell population, thus preventing disease relapse and ultimately improving patient survival. Suppression of Mcl-1 expression by the CDC7/CDK9 inhibitor PHA-767491 overcomes bone marrow stroma-mediated drug resistance in AML.	[110]
	Human bone-marrow-derived MSCs from ALL patients	Murine cell line MS-5; human bone-marrow-derived cells from patients with AML	N/A	Cytarabine	**Human MSCs are potent feeder cells that are able to maintain AML cells in long-term culture**. Co-culture of AML cells on MSCs results in a significantly higher proliferation capacity than on MS-5 or liquid culture. This favourable co-existence seems to be due, in part, to molecules important for communication within the niche. Blockade of TGF-β1 increases AML cell proliferation and chemosensitivity, while the CXCR4 antagonist plerixafor shows anti-proliferative effects and does not change cytarabine-induced cell death compared to control.	[105]
	Human MSCs from healthy volunteers	Human bone-marrow-derived and peripheral blood cells from AML patients	N/A	Cytarabine	Human MSCs contribute to quiescence and therapy resistance of persistent AML cells. Co-culture studies demonstrate that **hMSCs protect leukaemic cells from the effect of AraC (cytarabine) treatment by enriching quiescent cells**, mimicking the effects observed in patients. This effect is even detectable when no direct stromal contact is established.	[102]
	Human bone-marrow-derived MSCs from ALL patients	Leukaemia cell lines U937, HL60, and KG1a	NOD/SCID mice	Mitoxantrone	**MSC protects AML cells from apoptosis through the c-Myc-dependent pathway. The expression of microRNA-494 (miR-494) in AML cells after** **co-culture with MSCs is downregulated**. In the co-culture system, activation of miR-494 in AML cells suppresses proliferation and induces apoptosis of AML cells in vitro. After the addition of mitoxantrone to the co-culture system, the proliferation of AML cells with miR-494 activation is suppressed more than that of control cells. After subcutaneous injection of AML cell lines in combination with MSC, tumour growth is suppressed in mice injected with miR-494-overexpressing AML cells. The rate of tumour formation is even lower after mitoxantrone treatment in the miR-494-overexpressing group. miR-494 suppresses drug resistance in AML cells by downregulating c-Myc through interaction with MSCs.	[109]
	Human bone-marrow-derived MSCs from healthy volunteers	Leukaemia cell lines OCI-AML3, KG-1 cells, and Molm13	NSG mice	Cytarabine	**ARC enhances the migration and adhesion of leukaemia cells to MSCs** both in vitro and in a novel human extramedullary bone/bone marrow mouse model. ARC induces IL1β expression in AML cells and increases CCL2, CCL4, and CXCL12 expression in MSCs, both through ARC-mediated activation of NFκB. Cells from AML patients express the receptors for and migrate toward CCL2, CCL4, and CXCL12. **Inhibition of IL1β suppresses AML cell migration and sensitizes the cells co-cultured with MSCs to chemotherapy**.	[115]
	Human bone-marrow-derived MSCs from AML patients	AML cell line HEL; ossification cell line hFOB1.19	N/A	Daunorubicin	**A strong positive correlation between the thrombopoietin (TPO) level and c-MPL expression is found in the bone marrow mononuclear cells of relapsed AML patients**. A high level of TPO/c-MPL signalling may protect MSCs from daunorubicin chemotherapy in AML. The effects of inhibition of the TPO/c-MPL pathway on enhancing the chemotherapy sensitivity of AML cells and on their downstream effector molecules that direct the interactions between patient-derived blasts and leukaemia-repopulating cells need to be further studied.	[107]
**CLL**	Human bone-marrow-derived MSCs from CLL patients and healthy volunteers	Human peripheral-blood-derived cells from patients with CLL	N/A	Fludarabine, Cyclophosphamide, Bendamustine, Prednisone, and Hydrocortisone	**The presence of BM-MSCs rescues chronic lymphocytic leukaemia (CLL) cells from apoptosis** both spontaneously and following induction with various drugs, including fludarabine, cyclophosphamide, bendamustine, prednisone, and hydrocortisone. The treatment with a combination of anti-Notch-1, Notch-2, and Notch-4 antibodies or γ-secretase inhibitor XII reverts this protective effect by day 3, even in the presence of the above-mentioned drugs.	[116]
**Multiple myeloma**	Human bone-marrow-derived MSCs from patients with multiple myeloma and healthy volunteers	Human multiple myeloma cell lines U266 and RPMI-8226	N/A	Melphalan or doxorubicin	BM-MSCs derived from patients with multiple myeloma (MM-MSCs), but not from healthy subjects (NM-MSCs), protect MM cells against the cytotoxicity of two chemotherapeutic agents (melphalan and doxorubicin). The pyrrolidine dithiocarbamate (PDTC), a potent and specific inhibitor of NF-κB, reverses the protective effects of MM-MSCs on MM cells in response to chemotherapy-induced apoptosis. Moreover, blocking autophagy by CQ or 3MA induces I-κBα phosphorylation and consequently prevents MM-MSC-mediated NF-κB signalling activation in MM cells. The results highlight **the significance of MM-MSC-activated autophagy in MM chemotherapy resistance**.	[119]
**Glioma**	Human glioma-associated MSCs from patients	Human cell line U87MG; human primary glioblastoma cells	BALB/c-nu mice	Temozolomide	**Conditioned media of glioma-associated mesenchymal stromal/stem cells (gaMSCs) promotes the proliferation, migration, and chemotherapy resistance of glioma cells**. The increased expression of FOXS1 and the activation of the EMT process in glioma cells under gaMSC-conditioned media was detected. The relationship of FOXS1, EMT, and temozolomide resistance in glioma cells was demonstrated through the regulation of FOXS1 expression in vitro and in vivo.	[122]
**Glioblastoma**	Human umbilical cord perivascular cells	Human glioblastoma cell lines SNB-19 and U251	Chicken chorioallantoic membrane	Temozolomide	Human umbilical cord perivascular cells (HUCPVCs, an MSC population present in the Wharton’s jelly of the umbilical cord) secrete molecules that contribute to glioblastoma aggressiveness by increasing cell proliferation, migration, and viability in vitro and by stimulating higher tumour growth in vivo. In contrast, **the resistance of glioblastoma cells to temozolomide chemotherapy is not significantly affected by HUCPVC-conditioned medium**.	[124]
**Neuroblastoma**	Human bone-marrow-derived MSCs from patients with neuroblastoma	Human neuroblastoma cell lines CHLA-171 and CHLA-255	N/A	Etoposide, JAK inhibitor (AZD1480)	**Human bone marrow mesenchymal stromal cells induce tumour expression of sphingosine-1-phosphate receptor-1 (S1PR1), leading to their resistance to chemotherapy**. Targeting S1PR1 by shRNA markedly enhances etoposide-induced apoptosis in neuroblastoma (NB) cells, while overexpression of S1PR1 significantly protects NB cells from multidrug-induced apoptosis via activating JAK-STAT3 signalling. Treatment with FTY720, an FDA-approved drug and antagonist of S1PR1, dramatically sensitizes drug-resistant NB cells to etoposide in in vitro and human neuroblastoma xenograft models.	[125]
**Oral squamous cell carcinoma**	Human bone-marrow-derived MSCs	Human oral squamous cell carcinoma cell lines JHU-012, JHU-019, and OKF-TERT1	N/A	Cisplatin	**The crosstalk between human oral squamous carcinoma cells and MSCs is mediated, at least in part, by the activation of the autocrine PDGF-AA/PDGFR-α loop driving AKT-mediated signalling pathways**, resulting in reduced cancer cell sensitivity to cisplatin through alterations in apoptosis.	[129]
**Head and neck cancer**	Human bone-marrow-derived MSCs	Human head and neck cancer cell line SCC-25	BALB/c nude mice	Paclitaxel	MSC-exposed head and neck cancer cells develop paclitaxel resistance that can be maintained up to 30 d after the initial co-incubation period. **The secretory profile of the MSCs suggested IL-6 to be a potential mediator of epigenetic imprinting on the head and neck cancer cells**. When the MSC-imprinted cancer cells are exposed to the demethylation agent 5-aza-2′deoxycytidine, it restores the expression of the drug resistance genes to that of parental cells.	[130]
**Breast cancer**	Human bone-marrow-derived MSCs from healthy volunteers; Murine bone-marrow- or blood-derived MSCs	Human breast cancer cell line MDA-MB-231, human colon carcinoma cell line C26, human lung adenocarcinoma cell line LLC	BALB/c, C57Bl/6, athymic nude mice.	Cisplatin, irinotecan	**MSCs become activated during treatment with platinum analogues and secrete factors that protect cancer cells against a range of chemotherapeutics**. A metabolomics approach reveals two distinct platinum-induced polyunsaturated fatty acids (PIFAs), 12-oxo-5,8,10-heptadecatrienoic acid (KHT) and hexadeca-4,7,10,13-tetraenoic acid (16:4(n-3)), that in minute quantities, induce resistance to a broad spectrum of chemotherapeutic agents. Blocking central enzymes involved in the production of these PIFAs (cyclooxygenase-1 and thromboxane synthase) prevents MSC-induced resistance.	[154]
	Human bone-marrow-derived MSCs	Human breast cancer cell lines MDA-MB-231 and MCF-7	BALB/c female nude mice	Doxorubicin	Doxorubicin treatment induces the expression of miR-21-5p in MSCs and in mesenchymal stem-cell-derived exosomes, leading to the induction of S100A6 in the breast cancer cells (BCs). **Silencing of miR-21-5p expression in MSCs and MSC exosomes abolished the resistance of BCs to doxorubicin**, indicating an exosomal miR-21-5p regulated the role of S100A6 in chemoresistance both in vitro and in vivo.	[133]
	Human bone-marrow-derived MSCs	Human breast cancer cell line HCC1806	NOD/SCID mice	Mithramycin A, Doxycycline, 5-fluorouracil	When GFP-labelled BMMSCs and RFP-labelled HCC1806 cells are injected together in vivo, they create tumours that contain a new hybrid cell that has characteristics of both BMMSCs and HCC1806 cells. When hybrid cells are injected into the mammary fat pad of NOD/SCID mice, they produce xenograft tumours that are smaller in size and exhibit resistance to chemotherapy drugs (i.e., doxorubicin and 5-fluorouracil) compared tumours from HCC1806 cells alone. **This chemoresistance is shown to be associated with an increased expression of tetraspanins (CD9 and CD81) and drug resistance proteins (BCRP and MDR1)**. Subsequent siRNA-mediated knockdown of BMMSC-CD9 in DP-HCC1806:BMMSCs results in an attenuation of doxorubicin and 5-fluorouracil chemoresistance associated with decreased BCRP and serum cytokine expression (CCL5, CCR5, and CXCR12). **It is suggested that within the tumour microenvironment CD9 is responsible for the crosstalk between BMMSCs and HCC1806 breast cancer cells (via CCL5, CCR5, and CXCR12), which contributes to chemoresistance**.	[131]
	Bone-marrow-derived MSCs	Human breast cancer cell line MDA-MB-231	N/A	Valproic acid	In vitro experiments confirm that VA inhibits NF-kB activation in cancer cells. In addition, analysing gene expression data in patients taking oral valproic acid showed that this drug decreased the expression of antioxidant enzymes, culminating in increased oxidative stress in tumour cells. **Analysis of publicly available genome-wide drug-induced effects reveals that valproic acid, as a histone deacetylase inhibitor (HDACI), is the most effective drug in disturbing the signalling pathways activated by tumour–stromal interaction**.	[136]
	Human adipose-derived MSCs	Human breast cancer cell line MCF-7/ADR	N/A	Adriamycin hydrochloride	**Conditioned medium derived from adipose mesenchymal stem cells induces increased expression of C-terminal Src kinase (Csk)-binding protein (Cbp), accompanied by enhanced cell proliferation and chemotherapy resistance in MCF-7/ADR breast cancer cells**. Depletion of Cbp in breast cancer cells by RNA interference leads to remarkable inhibition of cell proliferation and invasion as well as synergy with adriamycin hydrochloride to suppress tumour growth.	[134]
	Human adipose-derived MSCs	Human breast cancer cell line MDA-MB-231	N/A	Doxorubicin	**Conditioned medium collected from hAdSCs elicits doxorubicin resistance and enhances the expression of ABCG2, which is a transporter responsible for the efflux of doxorubicin**. CXCL1 secreted by hAdSCs downregulates miR-106a expression in triple-negative breast cancer and thus upregulates the ABCG2 and doxorubicin resistance.	[132]
**Lung adenocarcinoma**	Human bone-marrow-derived MSCs from patients with non-haematological malignant tumours	Human lung adenocarcinoma cell lines H358, A549, and H460 and murine LLC cell line	C57BL/6 mice	Erlotinib	**Bone-marrow-derived MSCs residing in the hypoxic solid cancer microenvironment produce high levels of molecules associated with adipocytes, including adipokine leptin and IGFBPs. It is suggested that leptin induces the resistance of lung cancer cells to erlotinib through activating IGF-1R signalling**. IGFBP2 induces erlotinib resistance by activating IGF-1R signalling in an IGF-1-independent manner. IGFBP2 had a synergistic effect with leptin to induce erlotinib resistance in vitro and in vivo.	[137]
**Gastric cancer**	Human bone-marrow-derived MSCs	Human gastric cancer cell lines AGS and MKN45	BALB/C nude mice	FOLFOX regiment, composed of 5-FU, oxaliplatin, and calcium folinate	**MSCs promote stemness and chemoresistance in gastric cancer (GC) cells through fatty acid oxidation (FAO)**. TGF-β1 secreted by MSCs activates SMAD2/3 through TGF-β receptors, which then induce lncRNA MACC1-AS1 expression in GC cells and promote FAO-dependent stemness and chemoresistance through antagonizing miR145-5p. Pharmacologic inhibition of FAO with etomoxir (ETX) attenuates MSC-induced FOLFOX regiment resistance in vivo.	[141]
	Human gastric-cancer-associated MSCs from patients	The human GC cell lines SGC-7901, MGC-803, HGC-27, and AGS	BALB/c nude mice	5-fluorouracil	**Gastric cancer MSCs upregulate the levels of PD-L1 bound to the transcription factor CCCTC binding factor (CTCF), enhance the CSC-like** **properties of GC cells**, and lead to tumorigenesis. In vivo, PD-L1-positive GC cells have greater stemness potential and tumorigenicity than PD-L1-negative GC cells. GC cells are heterogeneous, and PD-L1s in GC cells have different reactivities to GCMSCs.	[138]
	Human bone-marrow-derived MSCs	Human gastric cancer cell lines AGS and MKN45, human renal epithelial cell line HEK293T	BALB/C nude mice	FOLFOX regiment, composed of 5-FU, oxaliplatin, and calcium folinate	MSC co-culture improves stemness and drug-resistance of gastric cancer (GC) cells. LncRNA histocompatibility leukocyte antigen complex P5 (HCP5) is induced in GC cells by MSC co-culture, contributing to stemness and drug resistance. **MSC-induced lncRNA HCP5 drives FAO through** **miR-3619-5p/AMPK/PGC1α/CEBPB axis to promote stemness and chemo-resistance of GC**.	[140]
	Human bone-marrow-derived MSCs	Human gastric cancer cell lines SGC7901, KATO-III, MKN45, and AGS	N/A	Cisplatin	**Bone marrow MSCs increase the antiapoptotic abilities and chemoresistance of CD133+ cells via upregulation of Bcl-2 and downregulation of BAX**. BM-MSCs triggered activation of the PI3K/AKT signalling cascade in CD133+ cells. Blocking the PI3K/AKT pathway inhibited the promotion of chemoresistance. BM-MSCs enhance the drug resistance of CD133-overexpressing cells in vitro and in vivo but not that of CD133-knockdown cells, which demonstrates the contribution of CD133 to this process.	[143]
	Human umbilical-cord-derived MSCs	Human gastric cancer cell lines HGC-27, MGC-803, and SGC-7901 and human foetal lung fibroblast cell line HFL1	BALB/c nu/nu mice	5-fluorouracil, cisplatin	MSC exosomes induce the resistance of gastric cancer cells to 5-fluorouracil both in vivo and ex vivo. **MSC exosomes antagonise 5-fluorouracil-induced apoptosis and enhance the expression of multi-drug-resistance-associated proteins**, including MDR, MRP, and LRP. **MSC exosomes trigger the activation of calcium/calmodulin-dependent protein kinases (CaMKs) and Raf/MEK/ERK kinase cascade** in gastric cancer cells. Blocking the CaM-Ks/Raf/MEK/ERK pathway inhibits the promoting role of MSC exosomes in chemoresistance.	[142]
**Pancreatic cancer**	Human bone-marrow-derived MSCs	Human pancreatic adenocarcinoma cell lines PANC1, MIA PaCa-2, and BxPC3, human glioblastoma cell line U-87MG, human colon carcinoma cell line HT29, human non-small-cell lung carcinoma cell line A549, human breast carcinoma cell line MCF7	SCID mice	Gemcitabine	The inhibiting of crosstalk between MSCs and tumour-initiating cells (TIC) disrupts the CXCL10–CXCR3 axis and sensitizes tumour cells to chemotherapy, mainly by targeting TICs residing in the treated tumour. **Based on the ability of MSCs to specifically home to tumours and target the TIC population, the use of MSC-derived nanovesicles as “Trojan horses”** is presented as a strategy to overcome resistance, especially in desmoplastic cancers such as pancreatic adenocarcinomas.	[144]
**Genitourinary cancers**	Human bone-marrow-derived MSCs	Human prostatic carcinoma cell lines PC3 and DU145	Nude mice	Docetaxel	MSCs reduce the sensitivity of castration-resistant prostate cancer (CRPC) cells to docetaxel-induced proliferation inhibition and apoptosis promotion in vivo and in vitro. **CRPC cells co-cultured with MSCs under docetaxel administration have an increased autophagy activation through TGF-β1 signalling. The autophagy inhibitor could effectively reverse MSC-induced resistance to docetaxel**, e.g., inhibition of TGF-β1 secretion in MSCs increases the sensitivity of CRPC cells to docetaxel.	[145]
	Human adipose-derived MSCs	Human epithelial ovarian carcinoma cell lines ES2 and SKOV3	N/A	Cisplatin	**Primary omental adipose-derived mesenchymal stem cells (ADSCs) are a contributor to cisplatin resistance, exhibiting an ability to reduce** **caspase-3-dependent apoptosis** and intracellular platinum accumulation in epithelial ovarian carcinoma EOC.	[147]
	Human amniotic-fluid-derived MSCs and adipose-derived MSCs from healthy volunteers	Human renal carcinoma cell line 786-0, human bladder carcinoma line T24	N/A	Ciprofloxacin	**MSC-conditioned medium reduces bladder and renal cancer cell viability** in vitro, **induces cell cycle perturbations** in bladder cancer T24 cells without significant influence on apoptosis rate in both studied cancer cell lines, and reduces cell sensitivity to ciprofloxacin after incubation in vitro with conditioned media.	[146]
	Human ovarian-cancer-associated MSCs from patients; human adipose-derived MSCs from healthy volunteers, human ovary-derived MSCs from healthy volunteers	Human ovarian cancer cell lines SKOV3, CAOV-3, COV318, Hey1, and PEO1	NOD/SCID mice	Cisplatin	Ovarian tumour-cell-secreted Hedgehog (HH) induces **CA-MSC BMP4 expression. CA-MSC-derived BMP4 reciprocally increases ovarian tumour cell HH expression, indicating a positive feedback loop**. Interruption of this loop with an HH pathway inhibitor or BMP4-blocking antibody decreases CA-MSC-derived BMP4 and tumour-derived HH, preventing enrichment of cancer stem-like cells (CSCs) and reversing chemotherapy resistance. The impact of HH inhibition is only seen in CA-MSC-containing tumours, indicating the importance of a humanized stroma. The results are reciprocal to findings in pancreatic and bladder cancer, suggesting HH signalling effects are tumour-tissue-specific, warranting careful investigation in each tumour type.	[148]
**Liver cancer**	Human MSCs	Human hepatocarcinoma cell lines SMMC-7721 and HepG2	N/A	3-methyladenine	Tumour inflammatory microenvironment is a key player in activating MSCs to induce chemoresistance of hepatocarcinoma cells. Inflammation is a fundamental feature during the development of hepatocellular carcinoma, which exists not only within the tumour tissue but also in the tissues surrounding the tumour. **MSCs in the inflammatory microenvironment may persistently promote the development of chemoresistance in HCC cells during tumour growth. One mechanism underlying the MSC-promoted development of chemoresistance in HCC cells is their overexpression of TGF-β in response to inflammatory stimuli in the tumour microenvironment**. Treatment of HCC cells with autophagy inhibitor effectively reverses the MSC-induced resistance to chemotherapy, and knockdown of TGF-β expression by MSCs with siRNA attenuates MSC-induced chemoresistance in HCC cells.	[151]
**Bone cancer**	Human bone-marrow-derived MSCs	Human osteosarcoma cell lines Saos-2 and U2-OS; primary cells from patients with osteosarcoma	BALB/c nude mice	Doxorubicin	**MSCs promote osteosarcoma cell survival and drug resistance through activation of STAT3**. Inhibition of STAT3 prolongs the survival time of tumour-bearing mice by suppressing tumour growth and increasing the sensitivity of tumour cells to doxorubicin. The increased expression of p-STAT3, multidrug resistance protein (MRP), and P-glycoprotein (MDR-1) is associated with high chemotherapy resistance in clinical osteosarcoma samples.	[152]

## 4. For a Successful Treatment, the Whole Tumour Microenvironment Must Be Considered: Crucial Findings

Based on the results of the studies presented in Chapter 3, we have identified crucial findings about the effect of MSCs on cancer cells, which are presented in the following subchapters. 

### 4.1. MSCs Affect Various Signalling Pathways in Cancer Cells to Protect Them from Chemotherapy-Induced Damage

Studies have shown that in the presence of chemotherapeutic agents MSCs in the tumour microenvironment play a protective role that prevents or at least decreases chemotherapy-induced damage in cancer cells. MSCs affect a large number of signalling pathways in cancer cells, most commonly those that protect cancer cells from apoptosis (e.g., increased expression of Bcl-2, Mcl-1, PGE2, ARC, and drug resistance proteins [110,114,115]) and promote their proliferation (e.g., altered expression of the Wnt, c-Myc, PI3K/AKT, and JAK2/STAT3 signalling pathways [99,106,108,125,155]). Moreover, these studies demonstrate the multifactorial ways in which the MSCs protect cancer cells and underline the reason for the difficulty in successfully preventing chemotherapy resistance and disease recurrence. 

### 4.2. MSCs Affect the Susceptibility of Cancer Cells to Chemotherapy When within the Tumour Microenvironment and Distant from the Tumour (Systemic Use)

While most of the studies included in this review article focused on the effects of MSCs or their conditioned medium in the tumour microenvironment, another study demonstrated that MSCs are not only capable of initiating a response within the tumour microenvironment but may also induce drug resistance when placed distant from the tumour. This was demonstrated by Roodhart et al. (2011), who first developed two mice tumour models using C26 colon carcinoma cells in BALB/c mice and Lewis lung carcinoma (LLC) cells in C57BL/6 mice. Next, they applied an intravenous injection of bone marrow MSCs at a distant site from the tumour at the time of cisplatin treatment, which led to complete resistance to the treatment in mice, which was mediated by the release of two platinum-induced fatty acids [154]. These were able to induce resistance against a broad spectrum of chemotherapeutic agents, especially platinum-containing chemotherapeutics. Interestingly, even the platinum-induced fatty acids alone (in the absence of MSCs) were able to induce resistance to chemotherapy, but the addition of COX-1 and thromboxane synthase to the MSC culture blocked the formation of chemotherapy resistance, as the production of the platinum-induced fatty acids depends on these enzymes [79]. 

### 4.3. How to Improve Treatment Outcomes

Successful cancer treatment without eventual disease relapse is extremely difficult to achieve due to the high complexity of the tumour microenvironment and the immense adaptability of cancer cells. However, several studies [30,96,99,106,116,119] demonstrated encouraging results when using inhibitors of various signalling pathways that are affected in cancer cells due to the presence and actions of MSCs in the tumour microenvironment. These results offer a good starting point for the investigation of novel treatment approaches and a much-needed shift from a cancer-cell-oriented treatment toward including the targeting of the stroma in the tumour microenvironment.

In the last years, many studies investigated the use of MSCs as a drug delivery tool for cancer treatment due to their ability to home to the tumour microenvironment. While there are still considerable challenges limiting effective translation, such as a limited tumour tropism and broad biodistribution when using conventional (non-altered) MSCs, the field is quickly developing [156,157,158]. However, one must consider the effect of the newly arrived MSCs on the tumour microenvironment after the delivery of chemotherapeutic agents. It is not clear whether the MSCs integrate into the tumour microenvironment and what effect they have on it, which raises crucial safety concerns. Hence, the studies included in this review article expose the hazards of bringing additional MSCs into the tumour microenvironment and underline the importance of thoroughly investigating the long-term effects of MSCs used as delivery tools.

## 5. Conclusions

The role of MSCs in the tumour microenvironment and the development of chemotherapy resistance has been quite well elucidated in the case of ALL, AML, and breast cancer. However, for most cancers, only few or even no studies investigating the role of MSCs in the development of resistance to chemotherapy have been published. Therefore, to ensure better treatment outcomes, future research should focus on the effect of non-cancerous cells, especially MSCs, in the tumour microenvironment on chemotherapeutic agents in all cancers and plan treatments in compliance with these results. 

Furthermore, discrepancies may arise when comparing results from the in vitro and in vivo settings, as MSCs can reduce cancer cell proliferation in the in vitro setting while promoting tumour growth in mice [38]. These results highlight the impact of the complex tumour microenvironment on cancer cells, which should be especially considered when using in vitro models that must contain multiple cell types in biologically relevant ratios. Furthermore, to obtain clear and solid data on the effects of non-cancerous cells in the tumour microenvironment, studies should include a combination of investigations in the in vitro and in vivo settings.

## Abbreviations

ALDH	Aldehyde dehydrogenase
ALL	Acute lymphocytic leukaemia
AML	Acute myeloid leukaemia
ARC	Apoptosis repressor with caspase recruitment domain
BDNF	Brain-derived neurotrophic factor
CXCL	Chemokine (C-X-C motif) ligand
CXCR	Chemokine (C-X-C motif) receptor
FGF-2	Fibroblast growth factor 2
HGF	Hepatocyte growth factor
ICAM-1	Intercellular adhesion molecule 1
IDO	Indoleamine 2,3-dioxygenase
IFNγ	Interferon γ
IGF-1	Insulin-like growth factor 1
IGFBP-2	Insulin-like growth factor-binding protein-2
IL-8	Interleukin-8
IGF-1R	Insulin-like growth factor 1 receptor
LIF	Leukaemia inhibitory factor
MAPK	Mitogen-activated protein kinase
Mcl-1	Myeloid cell leukaemia -1
MCP-1	Monocyte chemotactic protein-1
M-CSF	Macrophage colony-stimulating factor
MIP-2	Macrophage inflammatory protein 2
MSCs	Mesenchymal stem/stromal cells
PGDF	Platelet-derived growth factor
PGE2	Prostaglandin E2
PI3K	Phosphoinositide 3-kinase
S1PR1	Sphingosine-1-phosphate receptor 1
SDF-1	Stromal cell-derived factor 1
STAT3	Signal transducer and activator of transcription 3
TGF-β	Transforming growth factor β
TNFα	Tumour necrosis factor α
VCAM-1	Vascular cell adhesion protein 1
VEGF	Vascular endothelial growth factor
VLA-4	Very late antigen-4
